# The Ser/Thr Phosphatase PP2A Regulatory Subunit *Widerborst* Inhibits Notch Signaling

**DOI:** 10.1371/journal.pone.0101884

**Published:** 2014-07-09

**Authors:** Anasua Bose, Adam T. Majot, Ashok P. Bidwai

**Affiliations:** Department of Biology, West Virginia University, Morgantown, West Virginia, United States of America; Indiana University, United States of America

## Abstract

Drosophila Enhancer of split M8, an effector of Notch signaling, is regulated by protein kinase CK2. The phosphatase PP2A is thought to play an opposing (inhibitory) role, but the identity of the regulatory subunit was unknown. The studies described here reveal a role for the PP2A regulatory subunit *widerborst* (*wdb*) in three developmental contexts; the bristle, wing and the R8 photoreceptors of the eye. *wdb* overexpression elicits bristle and wing defects akin to reduced Notch signaling, whereas hypomorphic mutations in this PP2A subunit elicit opposite effects. We have also evaluated *wdb* functions using mutations in *Notch* and *E(spl)* that affect the eye. We find that the eye and R8 defects of the well-known *N^spl^* mutation are enhanced by a hypomorphic allele of *wdb*, whereas they are strongly rescued by *wdb* overexpression. Similarly, ectopic *wdb* rescues the eye and R8 defects of the *E(spl)D* mutation, which affects the *m8* gene. In addition, *wdb* overexpression also rescues the bristle defects of ectopically expressed M8, or the eye and R8 defects of its CK2 phosphomimetic variant M8-S159D. The latter finding suggests that PP2A may target M8 at highly conserved residues in the vicinity of the CK2 site, whose phosphorylation controls repression of Atonal and the R8 fate. Together, the studies identify PP2A-Wdb as a participant in Notch signaling, and suggest that M8 activity is controlled by phosphorylation and dephosphorylation. The conservation of the phosphorylation sites between Drosophila E(spl) and the HES/HER proteins from mammals, reptiles, amphibians, birds and fish raises the prospect that this mode of regulation is widespread.

## Introduction

The Notch signaling pathway is highly conserved among metazoan organisms and plays a pivotal role in cell fate determination throughout development [1]–[4]. Extensive studies in invertebrate and vertebrate models have resulted in the identification of most of the components of this pathway, and revealed that Notch activation results in the transcription of a family of basic Helix-loop-Helix (bHLH) repressors [5]. These proteins, collectively called the Hairy-Enhancer of Split (HES) repressors, are the terminal effectors of Notch signaling [6]–[11].

Over the years a remarkably detailed picture has emerged on the conserved components and mechanisms controlling ligand binding, Notch receptor processing, the nuclear functions of its intracellular domain (NICD), and factors mediating expression of the HES repressors. Despite this progress, our understanding of the mechanisms by which the large number of HES repressors mediate the diverse functions of Notch still remains incomplete. Because of their conserved (bHLH) structure, it has been thought that the HES proteins are functionally redundant and that they act as dosage-dependent effectors of Notch signaling. Arguments against both views (see below) have emerged from studies in Drosophila, specifically during the patterning/selection of the ‘founding’ R8 photoreceptors in the compound eye and the bristle sensory organ precursors (SOPs).

The development of the R8 or SOP is dependent upon bHLH proneural activators [12], [13]. These are *atonal* (*ato*) in the case of the R8 photoreceptor [14], [15] and a group of activators encoded by the *achaete scute Complex* (*ASC*) in the case of the bristle SOP [16]–[21]. The expression of these activators maintains neural competency in groups of otherwise equipotential cells, the proneural clusters (PNCs). This broad expression of Ato/ASC is later refined by the HES repressors in a process called lateral inhibition [22]–[24], during which the presumptive R8/SOP activates Notch to elicit HES expression in all other cells of the PNC. The HES repressors then antagonize Ato/ASC, thereby ensuring the specification (birth) of a single R8/SOP from each PNC, which is critical for proper structure and patterning of the eye and bristles.

A body of genetic studies have sought to analyze the roles of the seven HES repressors of Drosophila encoded by the *Enhancer of split Complex*, *E(spl)C* [5], [25]–[37]. Although these studies were the first to identify the HES repressors and establish their role in tissue patterning, they have produced paradoxical results. For example, loss of the *E(spl)C* elicits the birth of supernumerary R8s/SOPs [38], phenotypes that mimic loss of the Notch receptor or the transcription factor *Suppressor of Hairless* (*Su(H)*) [39], [40], which controls E(spl) expression in concert with NICD. In contrast, gain-of-function (GoF) studies have elicited confounding results. While ectopic expression of most E(spl)-members extinguishes the bristle-SOP fate [31], [33], the R8 fate is largely refractory. This inactivity was observed even with eye-specific members such as E(spl)-M8 [41], whose expression correlates in time/space to the emergence of single R8 cells [42] and whose mutation (*E(spl)D*) elicits dominant loss of R8s and the eye [41], [43]. This was the first line of evidence that for E(spl)-M8, protein dosage is, by itself, insufficient to inhibit the R8 fate.

This paradox was resolved for E(spl)-M8 whose ability to bind and antagonize Ato requires phosphorylation by protein kinase CK2 [44]. This post-translational modification (PTM) converts autoinhibited (inactive) M8 to a conformation that is competent for binding and repressing Ato and the R8 fate [45]. CK2 targets Ser159 in a Ser-rich region of M8 (the P-domain), which is located in the C-terminal domain (CtD) and is highly conserved in Drosophila E(spl)-M8, -M5 and -M7, and in human HES6. Accordingly, CK2 phosphorylates HES6 within its similarly localized P-domain [46]. Like the M8-Ato interaction, phosphorylation is also key to the formation of a HES6-HES1 complex. This raises the likelihood that a better understanding of the regulation of M8 should reveal conserved mechanisms regulating HES repressors, and by extension Notch signaling. Because CK2 is required for cell viability, its roles have been evinced by RNAi or dominant-negative (DN) constructs [47], [48]. These studies reveal that reduced CK2 activity elicits twinned and juxtaposed R8s and SOPs, both hallmarks of impaired lateral inhibition, suggesting that regulation by PTM is likely to be more general to Notch-dependent resolution of the PNCs. A better understanding of this mode of regulation is warranted to fully appreciate mechanisms controlling Notch signaling.

The aforementioned studies, in turn, raise the question on control of E(spl)-M8 by dephosphorylation. A candidate enzyme is the phosphatase PP2A, whose role emerged in assays for impaired signaling in wild type and mutant *Notch* (*N^spl^*) backgrounds [48]. Specifically, increased dosage (GoF) of *microtubule star (mts)*, the unique catalytic subunit of Drosophila PP2A, elicits twinned R8s/SOPs, defects that closely mimic loss of Notch or CK2. The possibility thus arises that PP2A antagonizes Notch signaling. However, the participating PP2A regulatory subunit remained to be identified, and it was unknown if this phosphatase impacted E(spl)-M8 activity in vivo.

Drosophila PP2A, like the mammalian enzyme, is composed of a catalytic (C) and a scaffolding (A) subunit that associate with a regulatory (R) subunit [49]–[52]. The core (AC) dimer is ubiquitously expressed, but lacks target recognition due to a shallow active site. Target recognition (substrate-specificity) is conferred by regulatory subunits, which in Drosophila include *widerborst* (*wdb*), *twins* (*tws*), *B52* and *B56*. The studies described here focus on *wdb* [53]. Using mutations in *Notch* and *E(spl)*, we provide evidence that Notch signaling is potentiated by loss of *wdb* and antagonized by its overexpression, and that *wdb* mitigates the activity of ectopically expressed M8 protein. The multiple lines of evidence in the bristle, eye and wing identify *wdb* as a component of Notch signaling and suggest that repression by E(spl) proteins is controlled by phosphorylation and dephosphorylation.

## Materials and Methods

### Fly stock and crosses

Flies were raised at 24°C on standard Yeast-Glucose medium. All crosses were performed at 24°C, unless indicated otherwise. The Gal4 drivers were generously provided by other investigators or obtained from the Bloomington Stock Center (denoted by the prefix B). The Gal4 drivers are *109*-*68Gal4* (B6479), *scaGal4* [33], [54], and *E(spl)Gal4* (B8225). The following mutants were obtained from the Bloomington Stock Center; *wdb^KG02977^* (B12977), *N^55e11^, frt19A* (B28813), *N^spl^* (B118, B182) and *E(spl)D* (B2447). *wdb^EP3559^* (see ref [55]) flies were a gift from Amita Sehgal (U. Penn). The *UAS-m8* and *UAS-m8S159D* flies have been previously described by us [45]. The *UAS-RNAi* lines to the PP2A regulatory subunits were obtained from the Drosophila Genetic Resource Center and include *PP2A*–*B’^EY22564^* (Flybase #22569) and *tws^02414^* (Flybase #108872).

### Fly phenotypes

**Figure 1:**
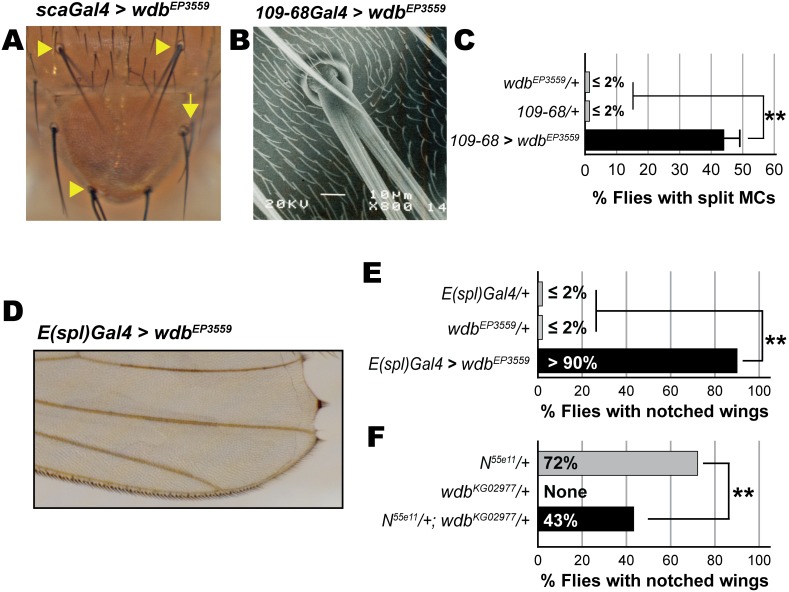
Wdb overexpression elicits bristle and wing margin defects. (A, B) Overexpression of Wdb elicits ectopic (arrow) and split macrochaetes (arrowhead). (B) Scanning EM of a split-MC. (C) Quantification of the penetrance of split-MCs; asterisks denote *P-values*<0.001 (n≥75). (D, E, F) Notched wing defects. (E) Overexpression of Wdb elicits wing margin defects. (F) Loss of *wdb* modifies the wing margin defects of *N^55e11^* flies. Asterisks in panels E and F denote *P-values*<0.001 (n≥44).

Fly heads were removed from newly eclosed flies. Heads were passed through a graded alcohol series for 24 hours each (25%–50%–75%-absolute), passed through Hexamethyldisalizane, and mounted on EM stubs using carbon tape (Ted Pella). Fly heads were dried for 24 hours, sputter coated with gold, and examined with a Hitachi scanning electron microscope at an accelerating voltage of 20 kV. Images were acquired as TIFF files that were collated using Adobe Illustrator. For quantitative analysis of eye size (facet number), crosses were established in duplicates, and ≥15 adult flies for each genotype were photographed using a Leica MZ16 stereomicroscope equipped with a Leica DFC-480 digital camera. Facet numbers were manually counted from TIFF images of the eyes and subjected to statistical analysis. For bristle phenotypes, newly eclosed adults were photographed using a Leica MZ16 stereomicroscope or by scanning EM. For quantitative analysis of the bristle phenotypes, multiple crosses were established (triplicates), and adults were scored for bristle defects. Wing margin phenotypes were documented using a Leica MZ16 stereomicroscope. Eye size (facet counts) and bristle and wing defects were statistically analyzed using the Student’s t-test, with the exception of Fig. 1F, which was determined using the chi-squared test.

### Immunostaining

Imaginal discs were isolated from late third instar larvae, fixed in freshly prepared 4% paraformaldehyde in 1x phosphate buffered saline (PBS) for 45 minutes at 4°C, and washed three times with PBS containing 0.3% Triton X-100 (PBS-TX). The discs were incubated for 12 hours at 4°C in PBS-TX containing 5% normal goat serum and primary antibody, washed three times with PBS containing 0.3% Triton X-100 (PBS-TX) and immunostained for 2–3 hours in secondary antibody. Following this, eye discs were washed three times with PBS containing 0.3% Triton X-100 (PBS-TX) and mounted in Vectashield. The following antibodies were used in this study; guinea pig anti-Sens (gift of Hugo Bellen, HHMI-Baylor) at a dilution of 1∶500 and mouse anti-ELAV (DSHB, Iowa) at a dilution of 1∶500. The mouse anti-ELAV antibody (9F8A9) developed by Gerald Rubin was obtained from the Developmental Studies Hybridoma Bank, created by the NICHD of the NIH and maintained at The University of Iowa, Department of Biology, Iowa City, IA 52242. Secondary antibodies (Molecular Probes) were goat-anti mouse-IgG coupled to Alexa Fluor 594 (1∶1000) and goat anti-guinea pig-IgG coupled to Alexa Flour 488 (1∶1000).

### Confocal microscopy

An Olympus FluoView (FV1000) was used for imaging. Images were generated from scans acquired every 1 µm along the apicobasal axis of the discs. Scanning was limited along the Z-axis to acquire full spectral output of the fluorophores, and no layers were removed from confocal stacks. Confocal images were compressed as a Z-stack, exported as TIFF files and collated in Adobe Illustrator.

## Results

### Increased *wdb* dosage elicits bristle and wing defects akin to Notch loss of function

Previous studies implicated the PP2A catalytic subunit, *mts*, in Notch signaling [48], but the relevant regulatory subunit remained to be identified. We focused on *wdb* because it had been identified in a GoF screen for genes that affect Drosophila bristle development [56]. This screen demonstrated that PNC-specific expression of *wdb^EP3559^* (*UAS-wdb*) by *scaGal4* elicits ectopic and split macrochaetes (MCs) on the notum (Fig. 1A), both suggesting reduced Notch signaling, but no follow-up studies have been reported on the underlying mechanism(s). The effects of *wdb^EP3559^* have been recapitulated by *UAS-wdb* constructs in other studies [53], [57], demonstrating the responsiveness of the *EP* insertion to the *GAL4-UAS* system.

Because *scaGal4/+* flies intrinsically display ectopic and split MCs with low-to-moderate penetrance (∼20–30%), we used the enhancer trap *sca^109-68^Gal4*, a driver of weaker strength (abbreviated as *109-68Gal4*, henceforth). *109*-*68Gal4/+* flies display a notably lower incidence of ectopic MCs (∼10%, data not shown), but importantly do not exhibit any split MCs (Fig. 1C). Using the latter as a bioassay, we find that ∼50% of flies overexpressing *wdb (wdb^EP3559^)* display split MCs (Fig. 1B, C). This defect requires *wdb* expression, as no split MCs are seen in *wdb^EP3559^/+* flies (Fig. 1C). Thus *wdb*-GoF elicits bristle defects akin to loss of Notch.

We sought to determine the role of Wdb in the developing wing margin. We first tested for *wdb*-GoF effects using *E(spl)Gal4*. We find that expression of *wdb* elicits notched wings with a high penetrance (Fig. 1D, E). The absence or extremely low incidence of notched wings in *wdb^EP3559^/+* flies or in *E(spl)Gal4/+* flies (Fig. 1E) reveals that this defect is *wdb*-dependent. The bristle and wing defects of *UAS-wdb* closely resemble those elicited by expression of *UAS-mts* with *109-68Gal4* or *E(spl)Gal4* [48]. We also assessed if loss of *wdb* would modify the classical wing margin defect of the Notch allele *N^55e11^*. Consistent with a *wdb*-GoF role in reducing Notch pathway activity, reduction of *wdb* dosage (through use of the hypomorphic allele *wdb^KG02977^*) yields a significant rescue of the wing margin defect of *N^55e11^* heterozygotes (Fig. 1F). No such wing margin defects are intrinsic to flies that are heterozygous or homozygous for *wdb^KG02977^* (Fig. 1F, and data not shown). Thus Wdb and Mts activities occur in similar developmental contexts and display effects consistent with reduced Notch signaling.

### *wdb* dosage modulates the retinal defects of *N^spl^*

Loss of CK2 compromises lateral inhibition and elicits ectopic (twinned) R8s and rough eyes [47]. If PP2A opposes Notch signaling, increased dosage of this phosphatase should elicit similar R8/eye defects. However, overexpression of *wdb* by *scaGal4* or *109*-*68Gal4* did not affect the R8s or the adult eye (data not shown, and see below), similar to the inactivity of ectopic Mts, reported by us [48]. This raised the possibility that the threshold requirement for PP2A is higher during R8 selection, compared to that in the bristle or wing (Fig. 1).

**Figure 2:**
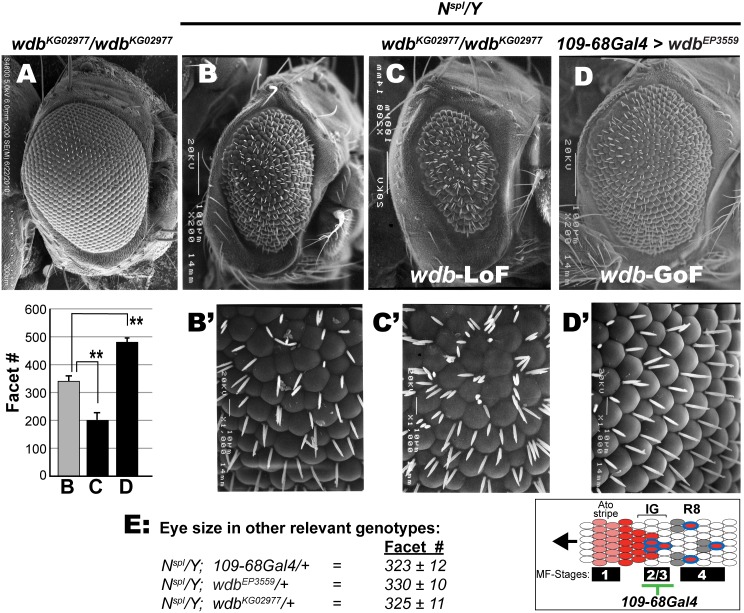
Modulation of the eye defects of N^spl^ by Wdb. Scanning EM of the adult eye at a magnification of 200x (A–D) and 1000x (B’–D’). (A) *wdb^KG02977^* homozygous flies display wild type eyes, whereas those of *N^spl^/Y* (males) are reduced and rough (B, B’). The *N^spl^* eye is exacerbated by *wdb^KG02977^* (C, C’), but rescued by overexpression of *UAS-wdb* (D, D’). Graph shows ommatidial (facet) counts of the eyes in panels B–D. Facet counts were determined in ≥15 flies, and asterisks denote P-values<0.001. (E) Facet counts in other relevant genotypes; note that wild type flies typically display 750–800 facets. Inset shows R8 selection from intermediate groups (IG). During R8 specification, Ato expression initiates at stage-1 of the MF, then upregulates, and finally resolves at stage-2/3, a region that coincides with the expression domain of *109*-*68Gal4*. Color codes are; Ato (pink/red), Sens (blue) and secondary R cells (grey).

To evaluate if *wdb* plays a role in R8 selection, we employed *N^spl^*, a widely studied mutation that mainly affects the eye. Specifically, *N^spl^* increases receptor sensitivity to the ligand Delta, thereby allowing inappropriate Notch activity in R8 precursors leading to loss of R8s [58]. Because morphogen secretion by differentiated R8s is required for *ato* expression and progression of eye development, further R8 specification becomes impaired. This reduces both, the number of ommatidia (facets) and their hexagonal phasing, which manifest as reduced and rough eyes, respectively (Fig. 2B, B’). Consistent with *N^spl^* rendering R8s sensitive to inhibitory Notch signaling, its eye defects are rescued by halved dosage of *Delta*, *Su(H)* or *E(spl)C* [42], [59], [60], or by conditions that should favor hypo-phosphorylation of M8 such as *CK2-RNAi* or Mts-GoF [43], [48]. This Notch mutation thus provides a sensitized background where modulation of endogenous E(spl) activity by CK2 or PP2A can be assessed.

Since the complete loss of PP2A elicits cell lethality [53], we tested if the hypomorphic allele *wdb^KG02977^* modifies the retinal defects of *N^spl^*. Indeed, when homozygous, *wdb^KG02977^* exacerbates the reduced and rough eye of *N^spl^* males (Fig. 2C, C’). This effect is not seen in *N^spl^/Y; wdb^KG02977^/+* flies (Fig. 2E) or in *wdb^KG02977^*/*wdb^KG02977^* flies (Fig. 2A), reflecting the hypomorphic nature of this *wdb* allele. In addition, mispatterning of the inter-ommatidial bristles (IOBs) was exacerbated in *N^spl^*/Y; *wdb^KG02977^*/*wdb^KG02977^* flies (compare Fig. 2B’ and 2C’), wherein multiple IOBs were found at normal and ectopic positions in the ommatidial lattice. On their own, homozygous *wdb^KGO2977^* flies do not exhibit such IOB defects (Fig. 2A). The effects of *wdb^KG02977^* reflect diminished PP2A activity, because precise excision of the P-element reverses the autophagy defects of this *wdb* mutation [57].

We next tested if the retinal defects of *N^spl^* are rescued by *wdb* overexpression. For this, we used the driver *109*-*68Gal4*, which is active at stage 2/3 of the morphogenetic furrow (MF, see [61], [62]) where lateral inhibition drives R8 selection (inset in Fig. 2). Indeed, overexpression of *UAS-wdb* (*wdb^EP3559^*) rescues the reduced eye of *N^spl^* (Fig. 2D), and markedly improves the hexagonal phasing of the ommatidia and the positioning of the IOBs (Fig. 2D’).

To provide quantitative analysis, we determined facet counts (graph in Fig. 2), an approach that has been used to compare eye size [43], [48], [63], [64]. The facet number for *N^spl^* males, 320±10, was used as a baseline. Consistent with the adult eye, *N^spl^*/Y; *wdb^KG02997^*/*wdb^KG02997^* flies display a significantly reduced facet count (∼200±11, see graph in Fig. 2), whereas overexpression of *wdb* significantly increased facet counts (∼480±11). Importantly, no modulation was seen in relevant control genotypes (Fig. 2E). Thus loss of *wdb* exacerbates *N^spl^*, whereas its overexpression rescues. The opposite effects of wdb-LoF and-GoF strengthen the role of this regulatory subunit in Notch signaling.

We also tested a recessive lethal allele of *twins*, *tws^02412^*, and B’ (*PP2A-B’^EY22564^*), but found that neither modulated *N^spl^* (data not shown). As an alternative, we expressed *UAS-RNAi* lines that target *tws* and the *B’* regulatory subunits with *109*-*68Gal4* but found that they did not modulate *N^spl^* (data not shown). Similarly, the recessive lethal allele *mts^XE2258^* did not exacerbate the reduced eye of *N^spl^* (data not shown), indicating that a 50% reduction in *tws* or *mts* activity is not limiting for PP2A activity. Therefore, our studies do not formally exclude the contributions of the regulatory subunits *tws* or *B’*.

### *wdb^KG02977^* enhances the R8 defects of *N^spl^*

**Figure 3:**
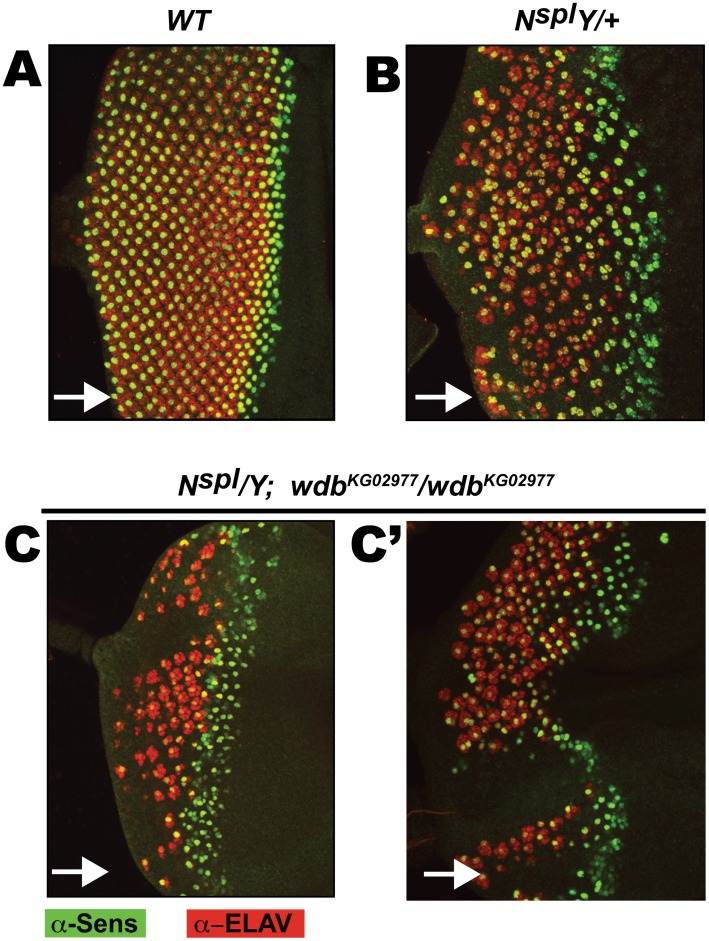
Loss of *wdb* activity enhances the R8 defects of *N^spl^*. Eye discs of the indicated genotypes were stained with α-Sens (Green) and α-ELAV (Red) to assess R8 differentiation and secondary photoreceptor recruitment, respectively. Panels C and C’ show two discs of the indicated genotype to illustrate random positioning of patches of non-specified and non-differentiated retinal tissue. Arrows denote direction of MF progression.

We next determined if the effects of *wdb^KG02977^* on *N^spl^* involve the R8 cells, the founding photoreceptors [65], [66]. For this, we stained third instar eye discs for Senseless (Sens), a marker for differentiated R8s [67], [68], and ELAV, a pan-neuronal marker [69]. In wild type discs (Fig. 3A) Sens expression initiates during R8 birth, after which Sens is maintained in differentiated R8s. To each R8, seven ELAV+ photoreceptors (R1–R7) are sequentially recruited. As previously shown, in *N^spl^* eye discs (Fig. 3B) Sens expression is not uniform along the dorso-ventral (DV) axis and is not sustained along the antero-posterior (AP) axis indicating that the inappropriate Notch activity impairs proper R8 differentiation. Importantly, *wdb^KG02977^* exacerbates the R8 defects of *N^spl^* (Fig. 3C), such that Sens expression is further diminished and poorly sustained along the AP axis. Notably, significant regions of the eye disc display no R8s or secondary photoreceptors. These patches of cells, which fail to undergo retinal fate specification, are randomly positioned in the eye disc (compare two such discs in Fig. 3C and C’) and do not manifest as gaps in the adult eye (see Fig. 2C), as unspecified cells are removed by apoptosis [70]. The reduced number and spacing of Sens+ and ELAV+ clusters are consistent with the exacerbated loss of the adult eye when *wdb* activity is compromised in *N^spl^* flies (Fig. 2C). Thus loss of *wdb* exacerbates the R8 defects of *N^spl^*.

### *wdb* overexpression rescues the eye defects of *N^spl^/+; E(spl)D/+* flies

**Figure 4:**
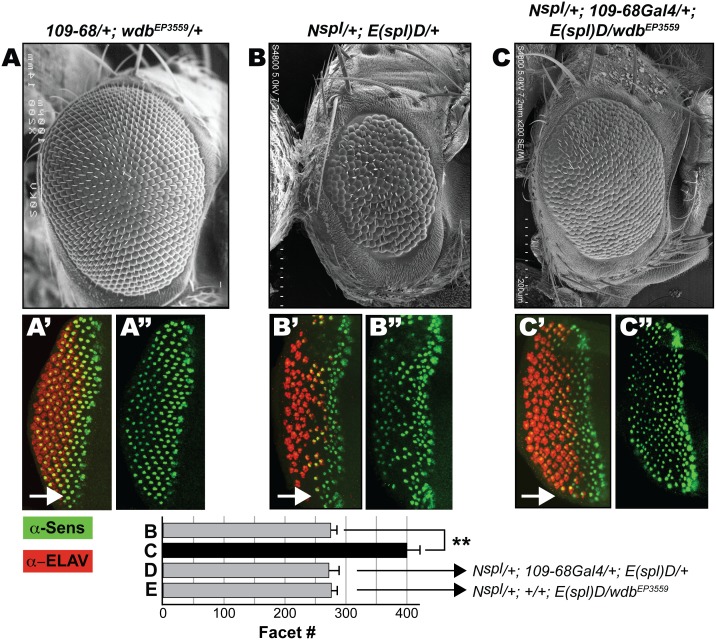
Ectopic Wdb rescues the eye defects of *N^spl^/+; E(spl)D/+* flies. Scanning EM of the adult eye at 200x magnification (A–C). (A) Overexpression of *wdb* does not perturb the adult eye. The reduced eye of *N^spl^/+; E(spl)D/+* flies (B) is rescued by overexpression of Wdb (C). Eye discs of the genotypes indicated in A–C were stained with α-Sens (Green) and α-ELAV (Red) to assess R8 differentiation and secondary photoreceptor recruitment, respectively, and arrows denote direction of MF progression. Panels A’–C’ show Sens+ELAV staining, whereas A’’–C’’ show Sens-only channel to highlight differentiated R8s. Graph shows ommatidial (facet) counts of the adult eyes. Data labeled B and C correspond to the adult eye shown in panels B and C. The genotype of relevant control progeny (D, E) is noted. Facet counts were determined in ≥15 flies, and asterisk denotes P-values<0.001.

We next evaluated the role of *wdb* in an *E(spl)* mutant background. We employed the dominant *m8* mutation *E(spl)D*, which elicits a reduced eye, but only in *N^spl^* flies (see Fig. 4B). Given the recessive nature of this Notch allele, *N^spl^/Y; E(spl)D/+* males display a severely reduced eye field (<15 facets, see ref. [41]) that is not amenable to modulation, whereas this effect is of moderate severity in *N^spl^/+; E(spl)D/+* females (∼275 facets, Fig. 4B). This moderate effect (in females) is amenable to modulation, given rescue by loss of function mutations in *Delta or E(spl)C* [42].

We find that overexpression of *wdb* with *109*-*68Gal4* rescues the reduced eye of *N^spl^/+; E(spl)D/+* females, appears to restore facet phasing (compare Fig. 4B and 4C), and significantly increases eye size (graph in Fig. 4). These effects require *wdb* expression, because no rescue is seen in the control genotypes (Fig. 4D, E). We next determined if rescue involves R8 specification and differentiation. Compared to WT (see Fig. 3A), *N^spl^/+; E(spl)D/+* eye discs display gaps between differentiated R8s, regions also devoid of secondary (ELAV+) photoreceptors (Fig. 4B’, B’’). Moreover, many of the R8s that are born fail to properly recruit ELAV+ cells, indicating that the progressive recruitment of secondary photoreceptors is impaired. These R8 defects are strongly rescued by overexpression of *wdb* in *N^spl^/+; E(spl)D/+* females (Fig. 4C’, C’’), an outcome consistent with the adult eye (Fig. 4C). Overexpression of *wdb* on its own does not affect the eye, R8 specification or secondary photoreceptor recruitment (Fig. 4A, A’, A’’). Thus increased *wdb* dosage rescues the eye and R8 defects of *N^spl^*, both alone or in combination with *E(spl)D*.

### *wdb* overexpression rescues the IOB loss of ectopic E(spl)-M8

**Figure 5:**
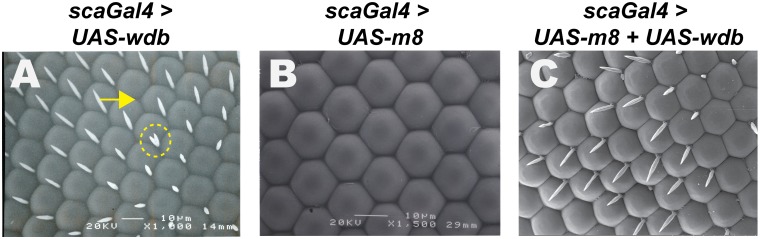
Ectopic Wdb rescues the IOB defects of E(spl)-M8. *UAS*-constructs were expressed with *scaGal4* and adult eyes were analyzed by scanning EM. Note that overexpression of Wdb alone (A) elicits occasional loss of IOB’s (arrow) or duplication (dotted circle). Overexpression of wild type M8 elicits strong loss of the IOBs, but does not perturb the hexagonal pattern of ommatidia (B). Co-expression of Wdb strongly rescues the IOB-loss of M8 (C).

We next tested if *wdb* overexpression would attenuate the activity of ectopic M8, as this would provide in vivo evidence that PP2A affects E(spl) activity, rather than expression of endogenous E(spl) proteins by perturbed Notch signaling. This was deemed important, because the modulation observed in our studies (Figs. 1–4) could formally result from altered E(spl) expression in response to *wdb*-GoF/LoF. We have previously shown that expression of *UAS-M8* (wild type) with *scaGal4* elicits strong loss of the IOBs, but does not affect R8 birth, eye size or facet phasing (Fig. 5B, see ref. [45]). Indeed, co-expression of *UAS-wdb* significantly restores IOBs (Fig. 5C). This effect is not seen upon co-expression of *UAS-LacZ* (data not shown), excluding competition for limiting amounts of Gal4 protein. Expression of *wdb*-alone elicits only occasional loss or duplicated IOBs (see Fig. 5A). As these studies were conducted in *Notch^+^* flies and M8 expression is under *Gal4-UAS* control, it seems unlikely that Wdb has direct (inhibitory) effects on components of the Notch receptor pathway.

### *wdb* overexpression rescues the eye defects of the CK2 mimic M8-S^159^D

**Figure 6:**
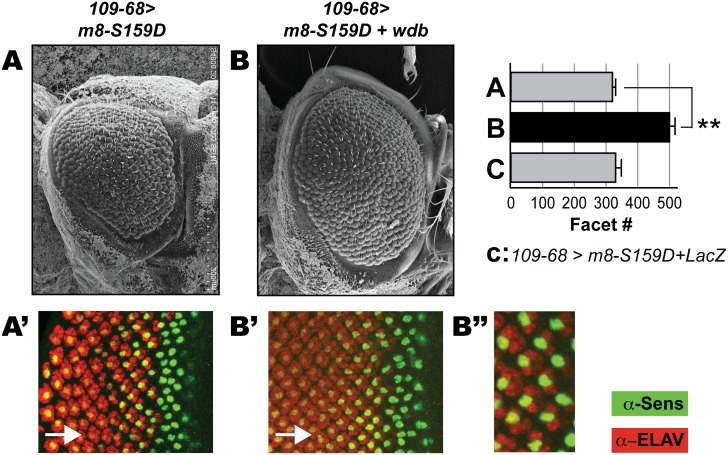
Ectopic Wdb rescues the eye defects of the CK2 mimic M8-S159D. Scanning EM of the adult eye at 200x magnification (A–B). (A) Overexpression of M8-S159D elicits a moderately reduced eye (A), which is strongly rescued by co-expression of Wdb. Graph shows ommatidial (facet) counts of the adult eyes. Data labeled A and B correspond to the adult eye shown in panels A and B. The genotype of relevant control progeny (C) is noted. Facet counts were determined in ≥15 flies, and asterisk denotes P-values<0.001. Eye discs of the genotypes indicated in A–B were stained with α-Sens (Green) and α-ELAV (Red) to assess R8 differentiation and secondary photoreceptor recruitment, respectively, and arrows denote direction of MF progression. Panels A’–B’ show Sens+ELAV staining, whereas B’’ show a magnification of B’ to highlight rescue of differentiated R8s and ELAV clusters.

We next tested if ectopic Wdb would rescue the reduced eye phenotype of a variant of M8 that harbors a phosphomimetic Asp in place of Ser159, a residue targeted by protein kinase CK2 [44]. This modification serves to activate M8 repression of Ato. We have previously reported [45] that expression of M8-S159D with *scaGal4* elicits a severely reduced eye (≤15 facets, likely a limit phenotype), whereas that with *109*-*68Gal4* is more muted (∼300 facets, Fig. 6A and graph). Since the effects with *109*-*68Gal4* are of moderate severity, we reasoned that these should be responsive to ectopic *wdb*. Indeed, co-expression of *wdb* rescued the reduced eye and significantly increased facet numbers (Fig. 6B and graph). As co-expression of *UAS-lacZ* is ineffective (Fig. 6 graph), rescue by ectopic *wdb* does not reflect competition for a limiting amount of Gal4. We next stained eye discs with Sens and ELAV. In *109*-*68/+; UAS-m8-S159D/+* eye discs, R8 patterning is perturbed, and these R8s poorly sustain Sens expression and inefficiently recruit secondary (ELAV+) photoreceptors (Fig. 6A’), as also observed in *N^spl^/+; E(spl)D/+* flies (see Fig. 4B’). These defects are rescued by co-expression of *wdb* (Fig. 6B’, see magnification in Fig. 6B’’). Importantly, in discs overexpressing M8-S159D+Wdb (Fig. 6B’, B’’), patterning of R8s appears closer to that in wild type discs (see Fig. 3A), which resemble those overexpressing only *wdb* (Fig. 4A’).

Together, the findings that ectopic Wdb rescues the eye/R8 defects of *N^spl^* males (Figs. 2, 3) or *N^spl^/+; E(spl)D/+* females (Fig. 4) support the possibility that Wdb enables PP2A to oppose Notch signaling, while rescue of the IOB defects of ectopic M8 (Fig. 5) or the eye/R8 defects of M8-S159D (Fig. 6) strengthen the likelihood that Wdb enables PP2A to target the Notch effector M8.

## Discussion

Control of protein activities by phosphorylation is a widespread regulatory mechanism, and reflects the need for rapid switching between two activity states. These modifications are often fine-tuned by the coordinated activities of phosphatases. One such enzyme is PP2A, whose role in Notch signaling was unclear. The studies described here identify *wdb* as a component of Notch signaling in multiple developmental contexts. We have conducted GoF and LoF studies on *wdb* in wild type flies, in *Notch* and *E(spl)* mutants, and upon ectopic expression of E(spl)-M8 or its CK2-site variant M8-S159D. Our studies suggest that PTM of HES repressors constitutes a sophisticated yet poorly studied component of Notch signaling.

Specifically, we demonstrate that ectopic *wdb* elicits bristle and wing margin defects that mimic loss of Notch (Fig. 1), rescues the eye/R8 defects of hypermorphic *Notch* and *E(spl)* mutations (Figs. 2, 3 and 4), and mitigates the activity of M8 during IOB development (Fig. 5) and that of the CK2 mimetic M8-S159D variant during eye/R8 development (Fig. 6). The effects of ectopic *wdb* are unlikely to reflect an artifact of mis-expression, because studies on *N^55e11^* (Fig. 1) and *N^spl^* (Figs. 2 and 3) show direct genetic interactions with the hypomorphic allele *wdb^KG02977^*. Importantly, the eye/R8 defects respond in a predictable manner, i.e., exacerbated by *wdb*-LoF but rescued by *wdb*-GoF. Together, the bristle, wing and eye/R8 analyses demonstrate that PP2A opposes Notch signaling, that this function is mediated by Wdb, and that this phosphatase mitigates the activity of E(spl)-M8, one of the bHLH effectors of this pathway.

It has been previously shown that decreased dosage of E(spl) or CK2 rescues the eye/R8 defects of *N^spl^*, as does an increase in the dosage of *mts*, the PP2A catalytic subunit. At face value, the studies of Figs. 2–4 could be the outcome if ectopic *wdb* were to inhibit the Notch receptor, components of this pathway or signaling to the nucleus, e.g., by impairing NICD or Su(H) activity. If any of these were the case, ectopic *wdb* would attenuate expression of endogenous E(spl) proteins or the mutant (M8) protein encoded by the *E(spl)D* allele (Fig. 4), either of which would elicit rescue. However, this possibility is diminished, because studies on the IOB defects of M8 (Fig. 5) and the eye and R8 defects of M8-S159D (Fig. 6) were in *Notch^+^* flies, and where the *Gal4-UAS* approach was used for transgene expression. Taken together, a simpler interpretation is that Wdb enables PP2A to mitigate E(spl)-M8 activity.

**Figure 7:**
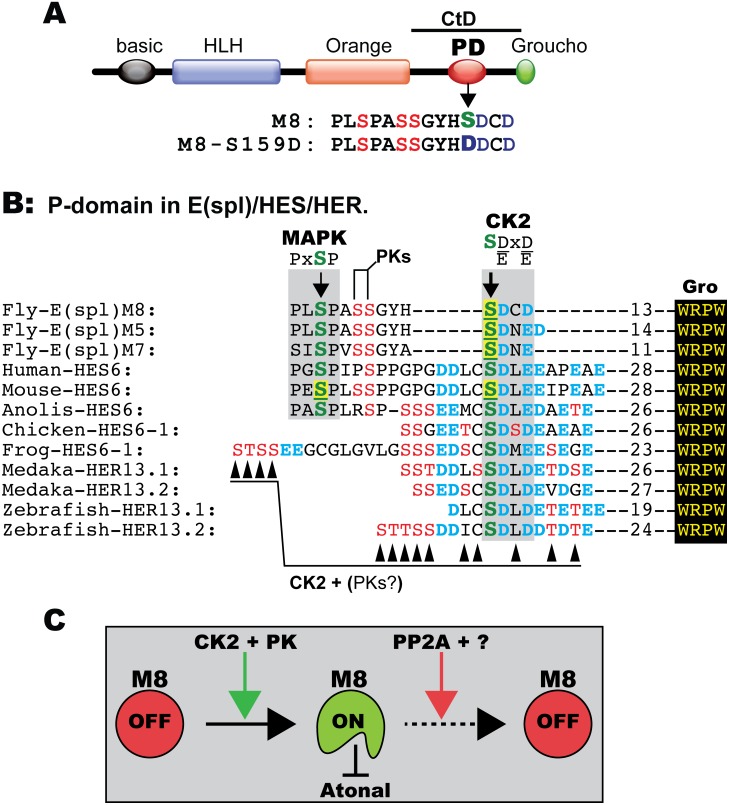
Conserved Ser residues in E(spl)/HES/HER and model for regulation of M8. (A) Functional domains in E(spl) proteins; the red oval is the Ser-rich phosphorylation domain (PD) located in the C-terminal domain (CtD). The P-domain of M8 and its CK2 mimetic form M8-S159D. (B) Alignment of the P-domain in Drosophila E(spl)-M8, -M5, -M7, HES6 from mammals/reptiles/birds/frogs, and the fish HER13 isoforms. The consensus sites for CK2 and MAPK are shown. Note the invariant CK2 site in E(spl)/HES/HER. The underlined Ser residues highlighted in yellow denote biochemically identified CK2 and MAPK sites. Arrowheads below alignment denote residues predicted to also be modified by CK2, and PK denotes yet unidentified protein kinases. The number of residues separating the P-domain from the C-terminal WRPW motif is indicated. (C) Model for regulation of M8. CK2 in concert with other protein kinases (PK) converts M8 into an active repressor of Atonal, whereas PP2A in concert with yet unidentified factors (?) mediate inactivation, through a conformational change or by destruction.

The modulation of the IOB defects of M8 (Fig. 5) appears more straightforward given that the P-domain is unperturbed (see Fig. 7A). In contrast, the modulation of the eye/R8 defects of the CK2 phospho-mimetic M8-S159D (Fig. 6) may seem paradoxical, as this Asp variant should not have been responsive to phosphatase activity if PP2A were to target the CK2 site. As shown in Fig. 7B, the P-domain is populated by a number of Ser residues that are highly conserved in M8, M7, M5, three of which are also conserved in the human, mouse and Anolis HES6. The importance of the CK2 site is now well understood for fly M8 and mouse HES6 (see Introduction), but the roles of the other highly conserved Ser residues are beginning to be resolved. In the case of HES6, the PG**S**P motif (see Fig. 7B) is targeted by MAPK [71], but its developmental role remains unknown. Likewise, the additional Ser residues in M8 appear to be subject to phosphorylation. In support, our ongoing studies reveal that replacement of the MAPK site of M8-S159D with PL**A**P neutralizes repression of Ato and the R8 fate (Bandyopadhyay and Bidwai, In preparation), raising the prospect that M8, like HES6, requires multi-site phosphorylation. In light of these findings, PP2A may target the MAPK site or modifications at the other Ser residues (see Fig. 7A), thereby controlling repressor activity. The possibility arises that coordinated functions of the participatory kinases and PP2A control M8 phosphorylation levels and/or activity (see model in Fig. 7C). Future biochemical studies are required to test this model for regulation, determine if Wdb permits PP2A to dephosphorylate M8, and identify which residue is a target of this phosphatase.

Remarkably, the CK2 Site Is Conserved and Similarly Located in Chicken/Frog HES6-1, as Well as in HER13.1/2 from Fish (Fig. 7B, and See Ref [72]). Even Though They Lack a Recognizable MAPK Site, the P-Domain in HES6-1/HER13.1/2 Harbors Several Conserved Ser/Thr Residues, Many of Which Meet the Consensus for CK2 (See Residues in Red in Fig. 7B). Because CK2 Is an Acidophilic Kinase That Utilizes Pser/Pthr as Surrogates for Asp/Glu [73]–[76], the Possibility Remains That HES6-1/HER13.1/2 Are More Extensively Phosphorylated by This Enzyme. This Possibility May Explain a Report [72] That Mutation of the Primary CK2 Site in Chicken HES6-1 (the Ser Residue Colored in Green in Fig. 7B) to Ala Does Not Affect Repression of HES5. Direct (in vitro) Biochemical Studies Are Needed to Determine If CK2 Targets a Single/Multiple Sites in HES6-1 and HER13.1/2.

The E(spl)/HES/HER proteins display length and sequence heterogeneity of the CtD. Computational analysis of fly E(spl) proteins reveals that the P-domain and its flanking sequences (aside from WRPW) are intrinsically disordered (ID), as is also the case for the HES/HER proteins (ref [64], and data not shown). It is becoming increasingly apparent that ID-regions, which are rich in Ser/Thr and Asp/Glu residues, are used to regulate activity through PTM [77]–[79]. The possibility thus arises that this region in E(spl)/HES/HER proteins serves as a ‘*charge-sensor*’ that controls activity via phosphorylation. If so, one might expect that evolutionary pressure would select for phosphorylatable residues, regardless of the kinases required. Such an argument has, in fact, been made for the circadian clock (see below), which requires multi-site phosphorylation of fly/human Period (PER, [80]) and the Frequency protein from Neurospora [81], even though the participatory kinases vary across taxa.

Our studies raise a number of questions relevant to our understanding of Notch signaling. We address each of these individually.

Firstly, how widespread is the regulation of E(spl)/HES proteins by PTM? The P-domain is highly conserved amongst M8, M7 and M5 (Fig. 7), and our recent studies have uncovered a variant P-domain in Mγ that is efficiently phosphorylated by CK2 (Jozwick and Bidwai, In preparation). Consequently, four E(spl) members are now known to be CK2-targets, whereas three (M3, Mβ and Mδ) appear to not be modified by this kinase. Importantly, the CK2 site in these four members is invariant in the homologous proteins from 12 Drosophila species that diverged over 50×10^6^ years (data not shown), a window of time over which mutations would have accumulated were these inconsequential for repressor activity.

Second, why are some E(spl)/HES proteins devoid of a P-domain? We speculate that this may reflect different modes by which E(spl)/HES proteins repress cell fate. Perhaps, the best example is the M8-Ato interaction. It was initially thought that E(spl) proteins bind N-box sequences to mediate repression [82]. However, no N-box has been identified in the *ato*-enhancers [83], leading to the view that protein-protein interactions underlie repression [84], [85]. In this model, heterodimers of Ato and Daughterless (Da) bind to E-boxes in the *ato*-enhancer [61], and repression by M8 involves formation of (DNA-binding independent) M8-Ato complexes. It is however, unlikely that this mode of repression is universal, given that the basic domain of HES members is conserved, and N-boxes have been identified in the *ASC* enhancer, whose mutations lead to impaired repression. Given the roles of HES proteins in myogenesis, oogenesis, somitogenesis, it is conceivable that either one or a combination of mechanisms regulate repression, or that in vivo targets of HES proteins are isoform-specific. Consistent with the latter possibility, the direct physical interaction of M8 with Ato has not been observed with E(spl)-Mγ and-Mδ [41], [86], two members whose expression in the MF closely correlates to birth of the R8s. Isoform specificity has also been reported for interactions of E(spl) members with ASC-bHLH activators [86].

Third, can the principles gleaned from other biological contexts apply to our studies on CK2, PP2A and Notch? The regulation of the central clock protein Period (PER) provides an attractive model. Detailed studies have revealed that PER activity/levels are regulated by CK2 and PP2A [55], [80], [87]–[89]. In this case, CK2 promotes PER activity, whereas PP2A opposes. Additional regulation is conferred by CK1 (*doubletime* [90]) and GSK3 (*shaggy* [91]), with PER degradation being controlled by CK1 and the F-box protein Slimb/β-TrCP [92], [93]. It is of interest to note that the additional Ser residues of the P-domain of M8 (Fig. 7A) resemble the consensus for CK1 and that phosphorylation is predicted to generate a strong site for Slimb binding (Majot and Bidwai, unpublished). If so, the cohort of kinases and phosphatases that regulate E(spl)-M8 may more closely resemble regulation of PER by PTM than has been recognized. Interestingly, sleep homeostasis is perturbed in *N^spl^* mutants [94], [95], although it is unknown if this reflects altered E(spl) expression and/or regulation. Future studies on E(spl)-M8 will be required to determine if its regulation is in fact similar to that of PER, or if the shared kinases and phosphatases is merely coincidental.

On its own, the shared regulation of M8 and HES6 makes for a strong evolutionary argument. Although the role of PP2A in HES6 regulation remains unknown, we note that HES6 regulates transitions in retinal cell fate specification and during development of the cerebral cortex, where progenitors give rise to the ordered specification of neurons (first), astrocytes (second) and oligodendrocytes (third) [96], [97]. It has, in fact, been proposed that regulated HES6 activity (phosphorylation/dephosphorylation) may underlie the timing of transitions from neurogenesis to astrocyte specification, during which HES6 promotes the former and inhibits the latter [71], [98]. In Drosophila, it may be the case that phosphorylation of M8 allows for proper R8 selection, whereas dephosphorylation controls rapid inactivation and/or clearance (see model in Fig. 7C). Given that R8 birth is closely followed (in time and space) by (Notch-dependent) recruitment of the R2/R5 photoreceptor pair, control by phosphorylation would be a significantly faster and more robust circuit, as compared to that based solely upon transcription. This mode of control would appear consistent with a mathematical model that R8 selection occurs in ≤10 minutes [99], a window of time more compatible with control of M8 activity/levels through PTM (phosphorylation and dephosphorylation). Such a mode of regulation may exploit tissue-specific expression patterns of select E(spl)-members [28], [100], their regulation by PTM and their preferred developmental targets.

Future studies to identify the site on M8 that is a target for PP2A, and whether HES6 is similarly regulated by dephosphorylation will be required to more fully reveal the mechanism(s) by which phosphorylation and dephosphorylation control repression. Thus, the observation of M8 control by PTM fortuitously opens a new window into regulation of Notch signaling, and raises the prospect that this mechanism is differentially employed in tissue patterning.

## References

[1] Artavanis-Tsakonas S, Muskavitch MA (2010) Notch: the past, the present, and the future. Curr Top Dev Biol 92: 1–29. 20816391 10.1016/S0070-2153(10)92001-2

[2] Ehebauer M, Hayward P, Arias AM (2006) Notch, a universal arbiter of cell fate decisions. Science 314: 1414–1415. 17138893 10.1126/science.1134042

[3] Tien AC, Rajan A, Bellen HJ (2009) A Notch updated. J Cell Biol 184: 621–629. 19255248 10.1083/jcb.200811141 PMC2686403

[4] Bray SJ (2006) Notch signalling: a simple pathway becomes complex. Nat Rev Mol Cell Biol 7: 678–689. 16921404 10.1038/nrm2009

[5] Delidakis C, Artavanis-Tsakonas S (1991) The enhancer of split [E(spl)] locus of Drosophila encodes seven independant helix-loop-helix proteins. Proc Natl Acad Sci U S A 89: 8731–8735. 10.1073/pnas.89.18.8731 PMC49994 1528887

[6] Sun H, Ghaffari S, Taneja R (2007) bHLH-Orange transcription factors in development and cancer. Transl Oncogenom 2: 105–118. 10.4137/tog.s436 PMC3634620 23641148

[7] Fisher A, Caudy M (1998) The function of hairy-related bHLH repressor proteins in cell fate decisions. Bioessays 20: 298–306. 9619101 10.1002/(SICI)1521-1878(199804)20:4<298::AID-BIES6>3.0.CO;2-M

[8] Dambly-Chaudiere C, Vervoort M (1998) The bHLH genes in neural development. Int J Dev Biol 42: 269–273. 9654008

[9] Bray SJ (1997) Expression and function of Enhancer of split bHLH proteins during Drosophila neurogenesis. Perspect Dev Neurobiol 4: 313–323. 9171445

[10] de Celis JF, de Celis J, Ligoxygakis P, Preiss A, Delidakis C, et al (1996) Functional relationship between Notch, Su(H) and the bHLH genes of the E(spl) complex: the E(spl) genes mediate only a subset of Notch activities during imaginal development. Develop 122: 2719–2928. 10.1242/dev.122.9.2719 8787746

[11] Jennings B, Preiss A, Delidakis C, Bray SJ (1994) The Notch signaling pathway is required for *Enhancer of split* bHLH protein expression during neurogenesis in Drosophila. Develop 120: 3537–3548. 10.1242/dev.120.12.3537 7821220

[12] Kiefer JC (2005) Proneural factors and neurogenesis. Dev Dyn 234: 808–813. 16059914 10.1002/dvdy.20522

[13] Bertrand N, Castro DS, Guillemot F (2002) Proneural genes and the specification of neural cell types. Nat Rev Neurosci 3: 517–530. 12094208 10.1038/nrn874

[14] Jarman A, Sun Y, Jan L, Jan Y (1995) Role of the proneural gene, atonal, in formation of Drosophila chordotonal organs and photoreceptors. Develop 121: 2019–2030. 10.1242/dev.121.7.2019 7635049

[15] Jarman AP, Grell EH, Ackerman L, Jan LY, Jan YN (1994) *atonal* is the proneural gene for Drosophila photoreceptors. Nature 369: 398–400. 8196767 10.1038/369398a0

[16] Calleja M, Renaud O, Usui K, Pistillo D, Morata G, et al (2002) How to pattern an epithelium: lessons from achaete-scute regulation on the notum of Drosophila. Gene 292: 1–12. 12119094 10.1016/s0378-1119(02)00628-5

[17] Modolell J, Campuzano S (1998) The achaete-scute complex as an integrating device. Int J Dev Biol 42: 275–282. 9654009

[18] Campos-Ortega JA (1998) The genetics of the *Drosophila achaete-scute* gene complex: a historical perspective. Int J Dev Biol 42: 291–297. 9654011

[19] Skeath JB, Carroll SB (1991) Regulation of achaete-scute gene expression and sensory organ pattern formation in the Drosophila wing. Genes & Dev 5: 984–995. 2044964 10.1101/gad.5.6.984

[20] Cubas P, de Celis JF, Campuzano S, Modolell J (1991) Proneural clusters of achaete-scute expression and the generation of sensory organs in the Drosophila imaginal wing disc. Genes & Dev 5: 996–1008. 2044965 10.1101/gad.5.6.996

[21] Ghysen A, Richelle J (1979) Determination of sensory bristles and pattern formation in Drosophila. II. The achaete-scute locus. Dev Biol 70: 438–452. 478169 10.1016/0012-1606(79)90037-x

[22] Axelrod JD (2010) Delivering the lateral inhibition punchline: it’s all about the timing. Sci Signal 3: pe38. 20978236 10.1126/scisignal.3145pe38

[23] Castro B, Barolo S, Bailey AM, Posakony JW (2005) Lateral inhibition in proneural clusters: cis-regulatory logic and default repression by Suppressor of Hairless. Develop 132: 3333–3344. 10.1242/dev.01920 15975935

[24] Simpson P (1990) Lateral inhibition and the development of the sensory bristles of the adult peripheral nervous system of Drosophila. Develop 109: 509–519. 10.1242/dev.109.3.509 2205467

[25] Preiss A, Hartley DA, Artavanis-Tsakonas S (1988) The molecular genetics of Enhancer of split, a gene required for embryonic neural development in Drosophila. EMBO J 7: 3917–3927. 3145200 10.1002/j.1460-2075.1988.tb03278.x PMC454979

[26] Knust E, Bremer KA, Vassin H, Ziemer A, Tepass U, et al (1987) The enhancer of split locus and neurogenesis in Drosophila melanogaster. Dev Biol 122: 262–273. 3109981 10.1016/0012-1606(87)90351-4

[27] Ziemer A, Tietze K, Knust E, Campos-Ortega JA (1988) Genetic analysis of Enhancer of split, a locus involved in neurogenesis in Drosophila melanogaster. Genetics 119: 63–74. 17246426 10.1093/genetics/119.1.63 PMC1203346

[28] Cooper MTD, Tyler DM, Furriols M, Chalkiadaki A, Delidakis C, et al (2000) Spatially Restricted Factors Cooperate with Notch in the Regulation of Enhancer of split Genes. Dev Biol 221: 390–403. 10790334 10.1006/dbio.2000.9691

[29] Heitzler P, Bourouis M, Ruel L, Carteret C, Simpson P (1996) Genes of the Enhancer of split and achaete-scute complexes are required for a regulatory loop between Notch and Delta during lateral signalling in Drosophila. Develop 122: 161–171. 10.1242/dev.122.1.161 8565827

[30] Jimenez G, Ish-Horowicz D (1997) A chimeric Enhancer of split transcriptional activator drives neural development and achaete-scute expression. Mol Cell Biol 17: 4355–4362. 9234693 10.1128/mcb.17.8.4355 PMC232289

[31] Nakao K, Campos-Ortega JA (1996) Persistent expression of genes of the Enhancer of Split Complex suppress neural development in Drosophila. Neuron 16: 275–286. 8789943 10.1016/s0896-6273(00)80046-x

[32] Dawson SR, Turner DL, Weintraub H, Parkhurst SM (1995) Specificity for the hairy/enhancer of split basic helix-loop-helix (bHLH) proteins maps outside the bHLH domain and suggests two separable modes of transcriptional repression. Mol Cell Biol 15: 6923–6931. 8524259 10.1128/mcb.15.12.6923 PMC230947

[33] Giebel B, Campos-Ortega JA (1997) Functional dissection of the Drosophila enhancer of split protein, a suppressor of neurogenesis. Proc Natl Acad Sci U S A 94: 6250–6254. 9177203 10.1073/pnas.94.12.6250 PMC21035

[34] Oellers N, Dehio M, Knust E (1994) bHLH proteins encoded by the Enhancer of split complex of Drosophila negatively interfere with transcriptional activation mediated by proneural genes. Mol Gen Genet 244: 465–473. 8078474 10.1007/BF00583897

[35] Klambt C, Knust E, Tietze K, Campos-Ortega JA (1989) Closely related transcripts encoded by the neurogenic gene complex Enhancer of split of Drosophila melanogaster. EMBO J 8: 203–210. 2540957 10.1002/j.1460-2075.1989.tb03365.x PMC400790

[36] Schrons H, Knust E, Campos-Ortega JA (1992) The enhancer of split complex and adjacent genes in the 96F region of Drosophila melanogaster are required for segregation of neural and epidermal cells. Genetics 132: 481–503. 1427039 10.1093/genetics/132.2.481 PMC1205151

[37] Knust E, Schrons H, Grawe F, Campos-Ortega JA (1992) Seven genes of the enhancer of split complex of Drosophila melanogaster encode helix-loop-helix proteins. Genetics 132: 505–518. 1427040 10.1093/genetics/132.2.505 PMC1205152

[38] Ligoxygakis P, Yu SY, Delidakis C, Baker NE (1998) A subset of Notch functions during Drosophila eye development require Su(H) and E(spl) gene complex. Develop 125: 2893–2900. 10.1242/dev.125.15.2893 9655811

[39] Schweisguth F (1995) Suppressor of Hairless is required for signal reception during lateral inhibition in the Drosophila pupal notum. Develop 121: 1875–1884. 10.1242/dev.121.6.1875 7601001

[40] Schweisguth F, Posakony JW (1992) Suppressor of hairless, the Drosophila homolog of the mouse recombination signal-binding protein gene, controls sensory organ cell fates. Cell 69: 1199–1212. 1617730 10.1016/0092-8674(92)90641-o

[41] Nagel A, Yu Y, Preiss A (1999) Enhancer of Split [E(spl)D] is a Gro-independent, hypermorphic mutation in Drosophila. Dev Genet 25: 168–179. 10440851 10.1002/(SICI)1520-6408(1999)25:2<168::AID-DVG11>3.0.CO;2-0

[42] Nagel AC, Preiss A (1999) Notch spl is deficient for inductive processes in the eye, and E(spl)D enhances split by interfering with proneural activity. Dev Biol 208: 406–415. 10191054 10.1006/dbio.1999.9203

[43] Kahali B, Bose A, Karandikar U, Bishop CP, Bidwai A (2009) On the mechanism underlying the divergent retinal and bristle defects of M8* (*E(spl)D*) in *Drosophila* . Genesis 47: 456–468. 19415625 10.1002/dvg.20521 PMC2777619

[44] Trott RL, Kalive M, Paroush Z, Bidwai AP (2001) Drosophila melanogaster casein kinase II interacts with and phosphorylates the basic-helix-loop-helix (bHLH) proteins m5, m7, and m8 derived from the Enhancer of split complex. J Biol Chem 276: 2159–2167. 11208814 10.1074/jbc.m005996200

[45] Karandikar U, Trott RL, Yin J, Bishop CP, Bidwai AP (2004) Drosophila CK2 regulates eye morphogenesis via phosphorylation of E(spl)M8. Mech Dev 121: 273–286. 15003630 10.1016/j.mod.2004.01.008

[46] Gratton M-O, Torban E, Jasmin SB, Theriault FM, German MS, et al (2003) Hes6 Promotes Cortical Neurogenesis and Inhibits Hes1 Transcription Repression Activity by Multiple Mechanisms. Mol Cell Biol 23: 6922–6935. 12972610 10.1128/MCB.23.19.6922-6935.2003 PMC193938

[47] Bose A, Kahali B, Zhang S, Lin J-M, Allada R, et al (2006) Drosophila CK2 regulates lateral-inhibition during eye and bristle development. Mech Dev 123: 649–664. 16930955 10.1016/j.mod.2006.07.003

[48] Kunttas-Tatli E, Bose A, Kahali B, Bishop CP, Bidwai AP (2009) Functional dissection of Timekeeper (Tik) implicates opposite roles for CK2 and PP2A during Drosophila neurogenesis. Genesis 47: 647–658. 19536808 10.1002/dvg.20543 PMC2917814

[49] Eichhorn PJ, Creyghton MP, Bernards R (2009) Protein phosphatase 2A regulatory subunits and cancer. Biochim Biophys Acta 1795: 1–15. 18588945 10.1016/j.bbcan.2008.05.005

[50] Janssens V, Goris J (2001) Protein phosphatase 2A: a highly regulated family of serine/threonine phosphatases implicated in cell growth and signalling. Biochem J 353: 417–439. 11171037 10.1042/0264-6021:3530417 PMC1221586

[51] Xu Y, Xing Y, Chen Y, Chao Y, Lin Z, et al (2006) Structure of the protein phosphatase 2A holoenzyme. Cell 127: 1239–1251. 17174897 10.1016/j.cell.2006.11.033

[52] Mumby M (2007) The 3D structure of protein phosphatase 2A: new insights into a ubiquitous regulator of cell signaling. ACS Chem Biol 2: 99–103. 17313179 10.1021/cb700021z

[53] Hannus M, Feiguin F, Heisenberg CP, Eaton S (2002) Planar cell polarization requires Widerborst, a B’ regulatory subunit of protein phosphatase 2A. Develop 129: 3493–3503. 10.1242/dev.129.14.3493 12091318

[54] Hinz U, Giebel B, Campos-ortega JA (1994) The basic-helix-loop-helix domain of Drosophila Lethal of Scute protein is sufficient for proneural function and activates neurogenic genes. Cell 76: 77–87. 8287481 10.1016/0092-8674(94)90174-0

[55] Sathyanarayanan S, Zheng X, Xiao R, Sehgal A (2004) Posttranslational regulation of Drosophila PERIOD protein by protein phosphatase 2A. Cell 116: 603–615. 14980226 10.1016/s0092-8674(04)00128-x

[56] Abdelilah-Seyfried S, Chan YM, Zeng C, Justice NJ, Younger-Shepherd S, et al (2000) A gain-of-function screen for genes that affect the development of the Drosophila adult external sensory organ. Genetics 155: 733–752. 10835395 10.1093/genetics/155.2.733 PMC1461115

[57] Banreti A, Lukacsovich T, Csikos G, Erdelyi M, Sass M (2012) PP2A regulates autophagy in two alternative ways in Drosophila. Autophagy 8: 623–636. 22330894 10.4161/auto.19081

[58] Li Y, Lei L, Irvine KD, Baker NE (2003) Notch activity in neural cells triggered by a mutant allele with altered glycosylation. Develop 130: 2829–2840. 10.1242/dev.00498 12756168

[59] Brand M, Campos-Ortega JA (1990) Second-site modifiers of the split mutation of Notch defines genes involved in neurogenesis in Drosophila melanogaster. Dev Genes Evol 198: 275–285. 10.1007/BF00377394 28305666

[60] Shepard SB, Broverman SA, Muskavitch MA (1989) A tripartite interaction among alleles of Notch, Delta, and Enhancer of split during imaginal development of Drosophila melanogaster. Genetics 122: 429–438. 2504643 10.1093/genetics/122.2.429 PMC1203714

[61] Powell LM, Zur Lage PI, Prentice DR, Senthinathan B, Jarman AP (2004) The proneural proteins Atonal and Scute regulate neural target genes through different E-box binding sites. Mol Cell Biol 24: 9517–9526. 15485919 10.1128/MCB.24.21.9517-9526.2004 PMC522279

[62] White N, Jarman A (2000) Drosophila atonal controls photoreceptor R8-specific properties and modulates both receptor tyrosine kinase and Hedgehog signalling. Develop 127: 1681–1689. 10.1242/dev.127.8.1681 10725244

[63] Jones C, Reifegerste R, Moses K (2006) Characterization of Drosophila mini-me, a Gene Required for Cell Proliferation and Survival. Genetics 173: 793–808. 16547096 10.1534/genetics.106.056762 PMC1526529

[64] Kahali B, Kim J, Karandikar U, Bishop CP, Bidwai AP (2010) Evidence that the C-terminal domain (CtD) autoinhibits neural repression by *Drosophila* E(spl)M8. Genesis 48: 44–55. 20014344 10.1002/dvg.20581

[65] Kumar JP (2012) Building an ommatidium one cell at a time. Dev Dyn 241: 136–149. 22174084 10.1002/dvdy.23707 PMC3427658

[66] Hsiung F, Moses K (2002) Retinal development in Drosophila: specifying the first neuron. Hum Mol Genet 11: 1207–1214. 12015280 10.1093/hmg/11.10.1207

[67] Nolo R, Abbott LA, Bellen HJ (2000) Senseless, a Zn finger transcription factor, is necessary and sufficient for sensory organ development in Drosophila. Cell 102: 349–362. 10975525 10.1016/s0092-8674(00)00040-4

[68] Frankfort BJ, Nolo R, Zhang Z, Bellen H, Mardon G (2001) senseless repression of rough is required for R8 photoreceptor differentiation in the developing Drosophila eye. Neuron 32: 403–414. 11709152 10.1016/s0896-6273(01)00480-9 PMC3122332

[69] Campos AR, Rosen DB, Robinow SN, White K (1987) Molecular analysis of the locus elav in Drosophila melanogaster: a gene whose embryonic expression is neural specific. EMBO J 6: 425–431. 3107982 10.1002/j.1460-2075.1987.tb04772.x PMC553413

[70] Wolff T, Ready D (1991) Cell death in normal and rough eye mutants of Drosophila. Develop 113: 825–839. 10.1242/dev.113.3.825 1821853

[71] Belanger-Jasmin S, Llamosas E, Tang Y, Joachim K, Osiceanu AM, et al (2007) Inhibition of cortical astrocyte differentiation by Hes6 requires amino- and carboxy-terminal motifs important for dimerization and phosphorylation. J Neurochem 103: 2022–2034. 17868320 10.1111/j.1471-4159.2007.04902.x

[72] Vilas-Boas F, Henrique D (2010) HES6-1 and HES6-2 function through different mechanisms during neuronal differentiation. PLoS One 5: e15459. 21151987 10.1371/journal.pone.0015459 PMC2996300

[73] Kuenzel EA, Krebs EG (1985) A synthetic substrate specific for casein kinase II. Proc Natl Acad Sci U S A 82: 737–741. 2983313 10.1073/pnas.82.3.737 PMC397121

[74] Kuenzel EA, Mulligan JA, Sommercorn J, Krebs EG (1987) Substrate specificity determinants for casein kinase II as deduced from studies with synthetic peptides. J Biol Chem 262: 9136–9140. 3474230

[75] Salvi M, Sarno S, Cesaro L, Nakamura H, Pinna LA (2009) Extraordinary pleiotropy of protein kinase CK2 revealed by weblogo phosphoproteome analysis. Biochim Biophys Acta 1793: 847–859. 19339213 10.1016/j.bbamcr.2009.01.013

[76] Litchfield DW (2003) Protein kinase CK2: structure, regulation and role in cellular decisions of life and death. Biochem J 369: 1–15. 12396231 10.1042/BJ20021469 PMC1223072

[77] Tompa P (2012) Intrinsically disordered proteins: a 10-year recap. Trends Biochem Sci 37: 509–516. 22989858 10.1016/j.tibs.2012.08.004

[78] Garza AS, Ahmad N, Kumar R (2009) Role of intrinsically disordered protein regions/domains in transcriptional regulation. Life Sci 84: 189–193. 19109982 10.1016/j.lfs.2008.12.002

[79] Dunker AK, Silman I, Uversky VN, Sussman JL (2008) Function and structure of inherently disordered proteins. Curr Opin Struct Biol 18: 756–764. 18952168 10.1016/j.sbi.2008.10.002

[80] Duvall LB, Taghert PH (2011) Circadian rhythms: biological clocks work in phospho-time. Curr Biol 21: R305–307. 21549947 10.1016/j.cub.2011.04.005

[81] Querfurth C, Diemfellner ACR, Gin E, Malzahn E, Hofer T, et al (2011) Circadian conformational change of the Neurospora clock protein FREQUENCY triggered by clustered hyperphosphorylation of a basic domain. Mol Cell 43: 713–722. 21884974 10.1016/j.molcel.2011.06.033

[82] Jennings BH, Tyler DM, Bray SJ (1999) Target Specificities of Drosophila Enhancer of split Basic Helix-Loop-Helix Proteins. Mol Cell Biol 19: 4600–4610. 10373509 10.1128/mcb.19.7.4600 PMC84258

[83] Sun Y, Jan L, Jan Y (1998) Transcriptional regulation of atonal during development of the Drosophila peripheral nervous system. Develop 125: 3731–3740. 10.1242/dev.125.18.3731 9716538

[84] Giagtzoglou N, Alifragis P, Koumbanakis KA, Delidakis C (2003) Two modes of recruitment of E(spl) repressors onto target genes. Develop 130: 259–270. 10.1242/dev.00206 12466194

[85] Baker NE (2004) Atonal points the way- protein-protein interactions and developmental biology. Dev Cell 7: 632–634. 15525524 10.1016/j.devcel.2004.10.010

[86] Alifragis P, Poortinga G, Parkhurst SM, Delidakis C (1997) A network of interacting transcriptional regulators involved in Drosophila neural fate specification revealed by the yeast two-hybrid system. Proc Natl Acad Sci U S A 94: 13099–13104. 9371806 10.1073/pnas.94.24.13099 PMC24269

[87] Blau J (2003) A new role for an old kinase: CK2 and the circadian clock. Nat Neurosci 6: 208–210. 12601377 10.1038/nn0303-208

[88] Akten B, Jauch E, Genova GK, Kim EY, Edery I, et al (2003) A role for CK2 in the Drosophila circadian oscillator. Nat Neurosc 6: 251–257. 10.1038/nn1007 12563262

[89] Lin JM, Kilman VL, Keegan K, Paddock B, Emery-Le M, et al (2002) A role for casein kinase 2alpha in the Drosophila circadian clock. Nature 420: 816–820. 12447397 10.1038/nature01235

[90] Preuss F, Fan JY, Kalive M, Bao S, Schuenemann E, et al (2004) Drosophila doubletime mutations which either shorten or lengthen the period of circadian rhythms decrease the protein kinase activity of casein kinase I. Mol Cell Biol. 24: 886–898. 10.1128/MCB.24.2.886-898.2004 PMC343813 14701759

[91] Martinek S, Inonog S, Manoukian AS, Young MW (2001) A role for the segment polarity gene shaggy/GSK-3 in the Drosophila circadian clock. Cell 105: 769–779. 11440719 10.1016/s0092-8674(01)00383-x

[92] Chiu JC, Vanselow JT, Kramer A, Edery I (2008) The phospho-occupancy of an atypical SLIMB-binding site on PERIOD that is phosphorylated by DOUBLETIME controls the pace of the clock. Genes Dev 22: 1758–1772. 18593878 10.1101/gad.1682708 PMC2492663

[93] Ko HW, Jiang J, Edery I (2002) Role for Slimb in the degradation of Drosophila Period protein phosphorylated by Doubletime. Nature 420: 673–678. 12442174 10.1038/nature01272

[94] Wu MN, Raizen DM (2011) Notch Signaling: A Role in Sleep and Stress. Curr Biol: R397–R398. 10.1016/j.cub.2011.04.014 21601800

[95] Seugnet L, Suzuki Y, Merlin G, Gottschalk L, Duntley SP, et al. (2011) Notch Signaling Modulates Sleep Homeostasis and Learning after Sleep Deprivation in Drosophila. Curr Biol. 10.1016/j.cub.2011.04.001 PMC3741064 21549599

[96] Koyano-Nakagawa N, Kim J, Anderson D, Kintner C (2000) Hes6 acts in a positive feedback loop with the neurogenins to promote neuronal differentiation. Develop 127: 4203–4216. 10.1242/dev.127.19.4203 10976052

[97] Bae S, Bessho Y, Hojo M, Kageyama R (2000) The bHLH gene Hes6, an inhibitor of Hes1, promotes neuronal differentiation. Develop 127: 2933–2943. 10.1242/dev.127.13.2933 10851137

[98] Jhas S, Ciura S, Belanger-Jasmin S, Dong Z, Llamosas E, et al (2006) Hes6 inhibits astrocyte differentiation and promotes neurogenesis through different mechanisms. J Neurosci 26: 11061–11071. 17065448 10.1523/JNEUROSCI.1358-06.2006 PMC6674651

[99] Lubensky DK, Pennington MW, Shraiman BI, Baker NE (2011) A dynamical model of ommatidial crystal formation. Proc Natl Acad Sci U S A 108: 11145–11150. 21690337 10.1073/pnas.1015302108 PMC3131319

[100] Wech I, Bray S, Delidakis C, Preiss A (1999) Distinct expression patterns of different enhancer of split bHLH genes during embryogenesis of Drosophila melanogaster. Dev Genes Evol 209: 370–375. 10370119 10.1007/s004270050266

